# Age‐associated variation in the gut microbiota of chinstrap penguins (*Pygoscelis antarctica*) reveals differences in food metabolism

**DOI:** 10.1002/mbo3.1190

**Published:** 2021-05-07

**Authors:** Jiashen Tian, Jing Du, Shengjiu Zhang, Yanqiu Li, Xianggang Gao, Jiabo Han, Zhichuang Lu

**Affiliations:** ^1^ Dalian Key Laboratory of Conservation Biology for Endangered Marine mammals Liaoning Ocean and Fisheries Science Research Institute Dalian China; ^2^ Dalian Sun Asia Tourism Holding Co., Ltd. Dalian China

**Keywords:** age, chinstrap penguin, gut microbiota, high‐throughput sequencing, metabolism, *Pygoscelis antarctica*

## Abstract

Age is known to affect the gut microbiota in various animals; however, this relationship is poorly understood in seabirds. We investigated the temporal succession of gut microbiota in captive chinstrap penguins of different ages using high‐throughput sequencing. The gut microbiota exhibited a significant age succession pattern, reaching maturity in adults and then declining with increasing age. Only 15 amplicon sequence variants were shared among the gut microbiota in chinstrap penguins at all studied ages, and these contributed to most of the age‐related variations in total gut microbiota. Co‐occurrence networks found that these key bacteria belonged to the genera *Acinetobacter*, *Clostridium* sensu stricto, and *Fusobacterium*, and more species interactions were found within the same taxonomy. Functional prediction indicated that most of the metabolic functions were more abundant in the gut microbiota in adult chinstrap penguins, except for carbohydrate metabolism, which was significantly more abundant in older individuals.

## Graphical abstract

The gut microbiota of chinstrap penguins exhibited a significant age succession pattern, reaching maturity in adults and then declining with increasing age. Only 15 bacterial strains were shared among the gut microbiota in chinstrap penguins at all studied ages, and these contributed to most of the age‐related variations in total gut microbiota. Most of the metabolic functions were more abundant in the gut microbiota of adult chinstrap penguins, except for carbohydrate metabolism, which was more abundant in older individuals.

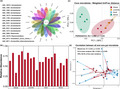

## 1 INTRODUCTION

The intestinal tract is an ecosystem in which bacteria and host cells interact to regulate food digestion and absorption by individuals (Zoetendal et al., 2004). It is therefore crucial to investigate the composition structure of the gut microbiota. However, although there is considerable information for mammals, studies on avian gut microbiota have mainly been carried out in poultry (Lan et al., 2005). Like all seabirds, penguins spend most of their lives at sea and come to land only when they are molting and breeding (Reilly, 1994; Stonehouse, 1975). Penguins can store large reserves of undigested food and build up large amounts of adipose tissue to help them long‐term survival during molting and breeding (Roeder et al., 2002). *Pygoscelis* includes three species, gentoo penguin (*P. papua*), Adélie penguin (*P. adeliae*), and chinstrap penguin (*P. antarctica*), all of which live in the cold ocean around the islands of Antarctica and the sub‐Antarctic (Vianna et al., 2017). Among these, *P*. *antarctica* is a specialist forager feeding almost exclusively on Antarctic krill (*Euphausia superba*) (Herman et al., 2017). Investigations of the gut microbiota in chinstrap penguins may thus provide an insight into the metabolism of seabirds with a specialist foraging ecology.

Although some bacteria may enter the eggs, the intestinal tract of birds is generally colonized by bacteria from different sources after hatching (Mills et al., 1999). Several factors, such as diet, climate, and the living environment, have been reported to introduce diverse bacteria into the gastrointestinal tract in birds (Lombardo et al., 1996). However, whether or not these bacteria colonize the gut depends on the physiological and genetic factors of the individuals. Among these factors, age can significantly impact the gut microbiota in various animals (Federica et al., 2018; Scott et al., 2017). For example, differences in gut microbiota induce by age have been found in some aquatic mammals, including the southern elephant seal (*Mirounga leonina*) (Nelson et al., 2013), Australian fur seal (*Arctocephalus pusillus doriferus*) (Smith et al., 2013), and spotted seal (*Phoca largha*) (Tian et al., 2020). Regarding birds, age‐related differences in gut microbiota have also been detected in tree swallows (Lombardo et al., 1996) and poultry (Scupham, 2007; Tanikawa et al., 2011). For penguins, current information on gut microbiota is still very limited. Using clone library technology, Banks et al., (2009) explored the effects of host phylogeny and geographical separation on the microbial composition of Adélie penguin guano. Information on the communities of gut microbiota in some Antarctic penguin species has been published for king, macaroni, gentoo, and little penguins (Dewar et al., 2013). In addition, the compositions of bacterial communities in stomach contents of Chinstrap and Adélie penguins were measured and compared (Yew et al., 2017). A previous study also reported on the age‐associated differences of gut microbiota in wild *P*. *antarctica*, but it only focused on adults and chicks (Barbosa et al., 2016). More importantly, the microbial composition in the gastrointestinal tract of little penguin throughput development was characterized using quantitative real‐time PCR and 16S rRNA gene sequencing (Dewar et al., 2017). There is thus a need to investigate the gut microbiota further to provide comprehensive insights into age‐related variations and their association with physiological functions in *P*. *antarctica*.

Throughout an animal's life, the bacterial composition of their intestinal tract may be affected by their social interactions, diet, and even stochastically mediated by the living environments (Palmer et al., 2007). It is therefore difficult to determine specific differences of the gut microbiota related to the age of hosts in natural environments. In contrast, captive animals that inhabit the same environment and feed on a consistent diet provide a suitable model for studying the variations of gut microbiota related to the age of hosts. In this study, we focused on analyzing the fecal microbiota, which closely reflects the assemblages present in the gastrointestinal tract without the need for invasive sampling (Meade, 1997). We determined the bacterial‐community compositions in the feces of captive *P*. *antarctica* of different ages in an aquarium in China using high‐throughput sequencing. The study aimed to obtain an overall view of the composition structure of the gut microbiota in captive *P*. *antarctica*, clarify the effects of age on the diversity and composition of gut microbiota, and explore the relationships between age‐associated variations in the gut microbiota of *P*. *antarctica* with their food metabolism and energy harvesting.

## 2 MATERIALS AND METHODS

### 2.1 Experimental animals and fecal collection

Fecal samples were collected from captive Chinstrap penguins in the Dalian Sun Asia Aquarium, China. Animals were living in an area measuring 30 × 7 m, with a water‐to‐land ratio of about 1:1, and a water depth of approximately 1.6 m. The ambient and water temperatures were 0°C and 7°C, respectively. These captive penguins were exclusively fed on capelin *Osmerus mordax*, accompanied by necessary mineral and vitamin supplements. We collected 20 fecal samples from 20 Chinstrap penguins of different ages within a single day. The penguins were classified as chicks (~2 years old), juveniles (7–8 years old), adults (18–21 years old), and seniors (25–26 years old). All the sampled penguins were healthy, and no antibiotics were used over the previous 3 months. Approximately 5 g wet weight of feces from each penguin was collected immediately after excretion with a sterilized container and stored at −80°C for further experiments.

### 2.2 DNA extraction and 16S rRNA ​gene sequencing

Bacterial genomic DNA from the fecal samples was extracted using a QIAamp PowerFecal DNA Kit (Qiagen, USA) in line with the instructions provided by the manufacturer. Successful DNA extraction was affirmed by electrophoresis with 1.5% agarose gel, and the purity and concentration of the extracted DNA were detected using a NanoDrop 2000 spectrophotometer (ThermoFisher, USA).

The V3–V4 regions of the 16S rRNA genes in each successfully extracted DNA sample were amplified using the primers 341F–806R, with unique barcodes and adapters added to the end of the reverse primer (Berg et al., 2012). All polymerase chain reactions (PCR) were executed in 15 μL of reaction mixture containing 7.5 μL of Phusion^®^ High‐Fidelity PCR Master Mix (New England Biolabs), 1 μL of dNTP (2.5 mM), 1 μL of forward and reverse primers (10 μM), and 1 μL template DNA. Thermal cycling consisted of initial denaturation at 95°C for 2 min, followed by 25 cycles of denaturation at 94°C for 5 s, annealing at 55°C for 30 s, and elongation at 72°C for 30 s (Zhao et al., 2020). The PCR products of each sample were detected by electrophoresis on 1.5% agarose gels. Then, the obtained PCR products were purified, quantified, and mixed in equal amounts to construct the sequencing libraries. The quality of the sequencing libraries was then determined using an Agilent Bioanalyzer 2100 system and Qubit^@^ 2.0 Fluorometer (Thermo Scientific, USA). Finally, the Illumina NovaSeq 6000 platform was applied to sequence these libraries with the 250 bp paired‐end strategy.

### 2.3 Sequencing data processing

Raw reads with ambiguous bases, average Phred score <20, homopolymer runs >6, mismatches in primers, or sequence length <150 bp were removed (Bokulich et al., 2013). The remaining clean reads were assigned to each sample according to the unique barcode combined with the end of the reverse primers. FLASH was used to assembled clean reads with an overlap >10 bp and without any mismatch into tags (Magoc & Salzberg, 2011). Then, the tags were qualified, assembled, and clustered into amplicon sequence variants (ASVs) using the DADA2 plug‐in unit in QIIME2 software (Bokulich et al., 2018). Each ASV was assigned to a taxonomy according to the SILVA database (Yilmaz et al., 2014), and an ASV abundance table was constructed. Singletons (number of tags in a specific ASV = 1) were discarded to improve the efficiency of data analysis (Tian et al., 2021), and the ASV table was normalized using a standard number of tags according to the sample with the least tags (Zhao et al., 2018). Finally, the functions of the gut microbiota were predicted using PICRUSt2 software (Douglas et al., 2020) based on the normalized ASV abundance table.

### 2.4 Statistical analysis

All analyses were conducted based on the normalized ASV abundance table. Alpha diversity indexes, including Chao1 and Shannon, were obtained using PAST v3.0 software (Hammer et al., 2001). The Chao1 index estimates the species richness based on a simple prediction of the species numbers (Colwell, 2009), and the Shannon index evaluates the diversity of the gut microbiota based on both the species counts and their relative abundances (Moreno & Rodríguez, 2010). Boxplots based on these diversity indices were created using R v4.0.2, and differences among *P*. *antarctica* of different ages were determined by Tukey's honest significant difference (HSD) test. Variations in the gut microbiota compositions of *P*. *antarctica* at different ages were assessed by PERMANOVA test and principal coordinate analysis (PCoA) using the “vegan” package in R v4.0.2, based on both unweighted and weighted UniFrac distances (Lozupone et al., 2011). Weighted Unifrac distance considers not only the species composition but also their relative abundance (Zhao et al., 2017), while unweighted Unifrac distance only considers the composition of the species, without their relative abundance (Zhang et al., 2020). Barplots based on the “ggplot2” package in R v4.0.2 software were used to visualize the relative abundances of dominant bacteria phyla and genera. In addition, the core microbiota among all studied *P*. *antarctica* was identified by Venn analysis, and variations in the core microbiota were compared by PCoA and the PERMANOVA test. The relative importance of the core microbiota to the variations in total gut microbiota was determined by variation partitioning analysis (VPA), Mantel test, and Procrustes analysis using the “vegan” package in R v4.0.2. Furthermore, differences among the gut microbiota of *P*. *antarctica* were deciphered by network analysis using the Molecular Ecological Network Analysis Pipeline (Deng et al., 2012). The detailed analytical process was executed according to a previous study (Ling et al., 2016), and the network graph was visualized with the Gephi software (Bastian & Jacomy, 2009). Finally, the predicted abundances of metabolism‐related pathways were exhibited by the “ggplot2” package in R v4.0.2, and differences among penguins of different ages were confirmed by Tukey's HSD test.

## 3 RESULTS

### 3.1 Summary of gut microbiota

**FIGURE 1:**
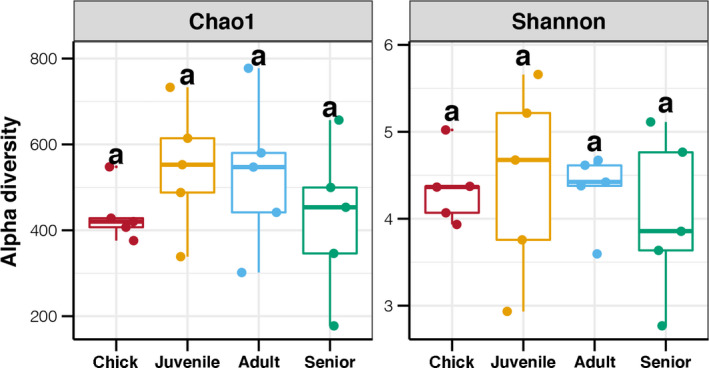
Age‐related differences in alpha diversity indices of gut microbiota in *P*. *antarctica*. Different lowercase letters above each box in the same subfigure represent significant differences between groups (Tukey's HSD test, *p* < 0.05)

We examined the diversity and composition of gut microbiota in captive *P*. *antarctica* individuals over a broad age range. Sequencing of the 20 fecal samples produced 2,822,368 sequences, with an average of 110,591 high‐quality tags per sample. The obtained tags were clustered into 6,373 ASVs, which were assigned to 98–521 genera in different samples. The alpha diversity indices of the gut microbiota were calculated, and differences among age groups were compared (Figure 1). The richness of the *P*. *antarctica* gut microbiota ranged from 177–732. There was a slight increase from chicks to adults, followed by a decrease with subsequent increases in age. However, no significant difference was found in the richness of gut microbiota among the different age groups (*p* > 0.05). In terms of gut microbiota diversity, there was no obvious variation in the Shannon index across the different age groups. Overall, these results suggest that, although some novel bacteria may have entered the gastrointestinal tract of *P*. *antarctica* throughout their life, these could not significantly affect the richness and evenness of the gut microbiota.

### 3.2 Age‐related differences in the gut microbiota of Chinstrap penguins

**FIGURE 2:**
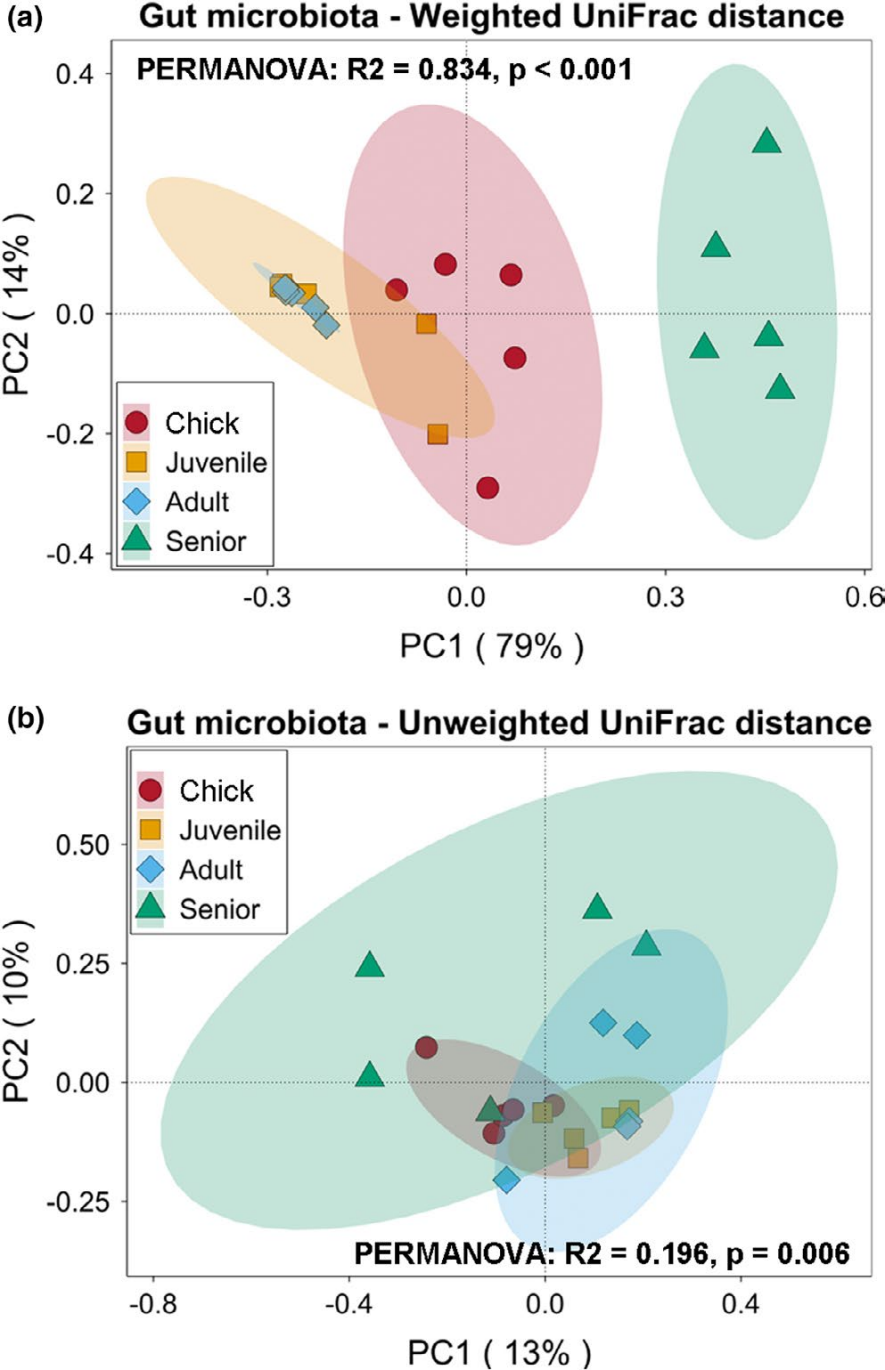
Principal coordinate analysis (PCoA) and PERMANOVA test of gut microbiota in *P*. *antarctica* of different ages based on weighted (a) and unweighted (b) UniFrac distances

The overall gut microbiota composition changed significantly with increasing age in *P*. *antarctica* (PERMANOVA test, *p* < 0.05, Figure 2). Based on the weighted UniFrac distance, age explained 83.41% of the variations in gut microbiota (Figure 2b), while the unweighted UniFrac distance only accounted for 19.6% (Figure 2a). According to the PCoA based on the weighted UniFrac distance, the gut microbiota of *P*. *antarctica* could be clustered into three groups based on the ages of hosts. Samples from juveniles and adults were more similar, and PC1 separated them away from the other samples, which explained 79% of the total variation (Figure 2a). By contrast, the first two principal components of PCoA based on the unweighted UniFrac distance only explained 23% of the total variation (Figure 2b). Moreover, within‐group differences in gut microbiota in relation to increasing age showed an initially decreasing trend followed by an increase, with the lowest intra‐group differences in adults. In contrast, PCoA of intra‐group differences in gut microbiota based on unweighted UniFrac distances revealed an increasing trend with increasing age of *P*. *antarctica* (Figure 2b).

### 3.3 Differences in relative abundances of intestinal bacteria

**FIGURE 3:**
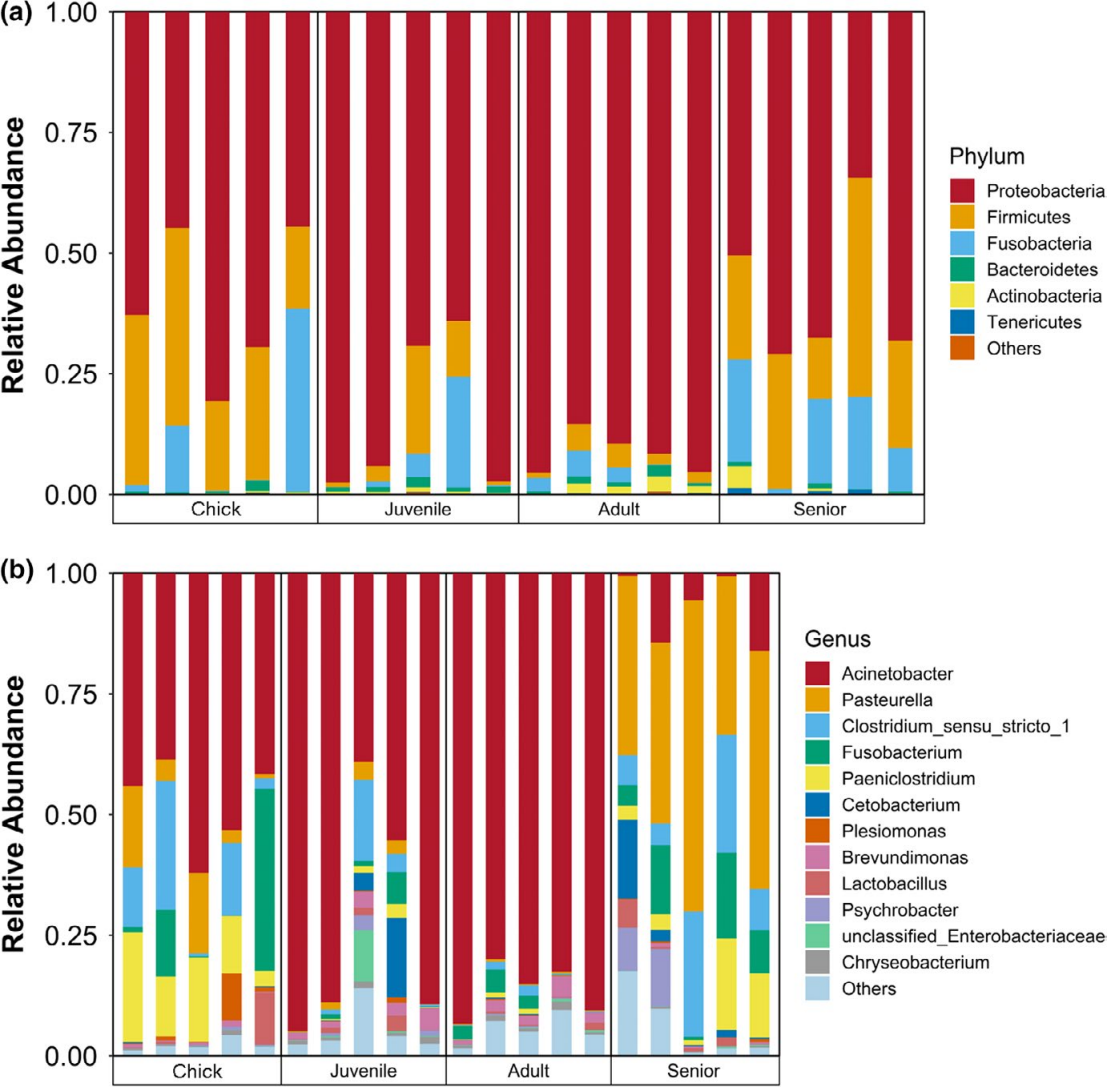
Relative abundances of dominant phyla (a) and genera (b) in the gut microbiota of *P. antarctica*

The relative abundances of dominant bacteria phyla in the gut microbiota of *P*. *antarctica* are shown in Figure 3a. Most microbes in all samples belonged to the phylum *Proteobacteria*, followed by *Firmicutes*, *Fusobacteria*, and *Bacteroidetes*. Among these, a significantly higher abundance of *Proteobacteria* was found in juveniles and adults than in chicks and seniors (*p* < 0.05). In contrast, *Firmicutes*, as the second most abundant phylum, were more abundant in chicks and seniors (*p* < 0.05). We further explored the age‐related differences in intestinal bacteria genera of *P*. *antarctica* (Figure 3b). *Acinetobacter* was the main genus of *Proteobacteria* in the gut microbiota in chicks, juveniles, and adults, whereas *Pasteurella* was the main genus in seniors. Meanwhile, *Paeniclostridium* and *Clostridium* sensu stricto were the dominant genera belong to the *Firmicutes* phylum, and *Fusobacterium* was the main genus of *Fusobacteria*.

### 3.4 Variations in core microbiota

**FIGURE 4:**
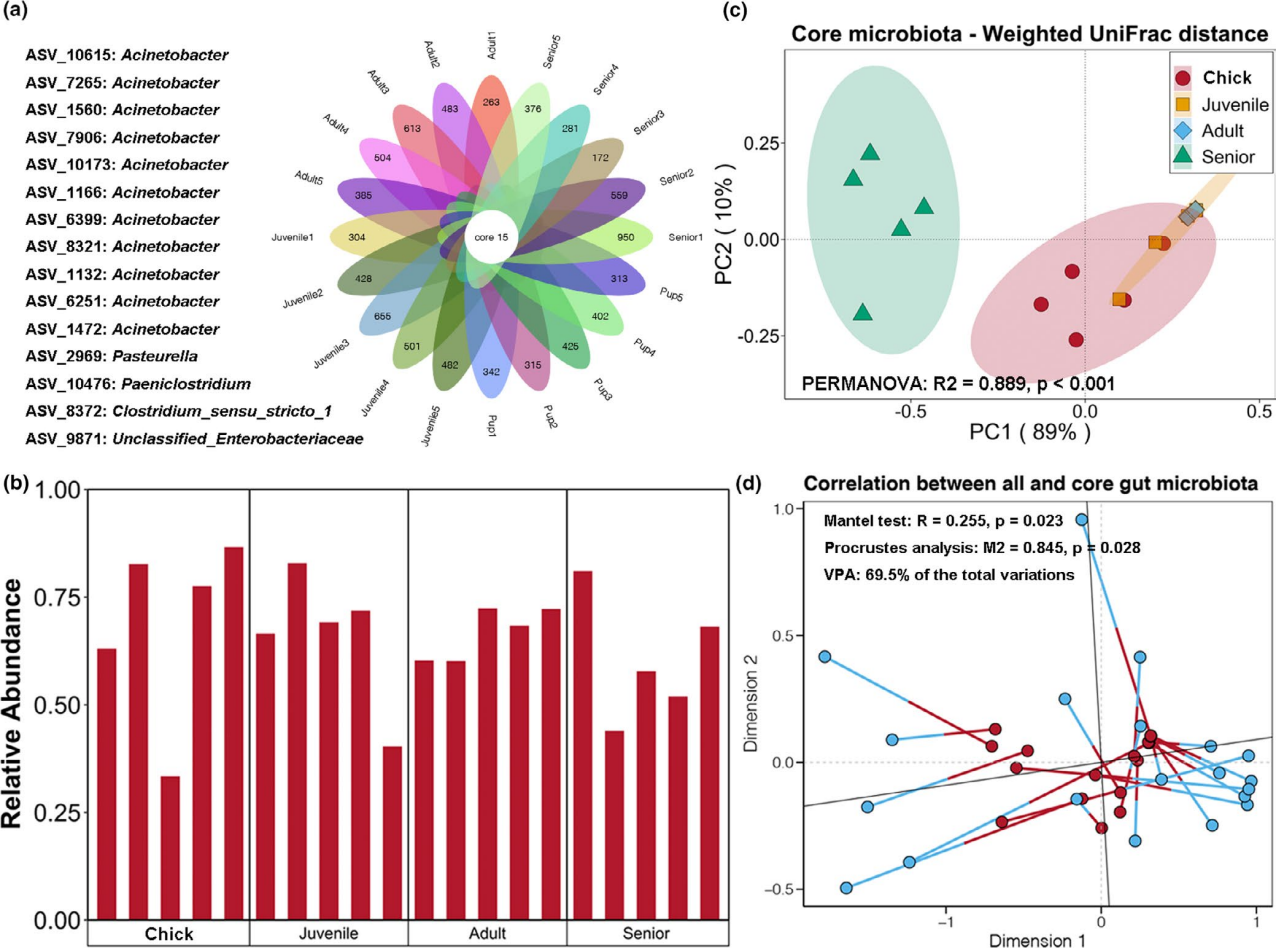
(a) Core ASV shared in all samples. (b) Relative abundance of core microbiota among all samples. (c) Principal coordinate analysis (PCoA) and PERMANOVA test of core microbiota in *P*. *antarctica* of different ages based on weighted UniFrac distance. (d) Mantel test, Procrustes analysis, and variation partitioning analysis revealed correlation and contribution of core microbiota to overall variations in gut microbiota

Venn analysis showed that only 15 ASVs were shared across all studied samples, mostly *Acinetobacter*, *Pasteurella*, *Paeniclostridium*, *Clostridium* sensu stricto, and *Unclassified_Enterobacteriaceae* (Figure 4a). Although accounting for limited numbers of the core ASVs, they accounted for 30%–80% of the total gut microbiota, with no significant differences in abundances among the different age groups (*p* < 0.05, Figure 4b). We also analyzed the variations in core microbiota among the different age groups based on the weighted UniFrac distance using PCoA and PERMANOVA. The results revealed that the core microbiota compositions differed significantly among the different age groups (PERMANOVA test, *p* < 0.05). The clustering pattern of the core microbiota was similar to that of the total gut microbiota, and the first two PCs explained almost 100% of the variations (Figure 4c). Mantel test and Procrustes analysis indicated a significant correlation between variations in the core and total gut microbiota (Figure 4d). VPA also showed that variations in the core microbiota could explain 69.5% of the changes in the total gut microbiota among the different age groups.

### 3.5 Co‐occurrence networks of gut microbiota

**FIGURE 5:**
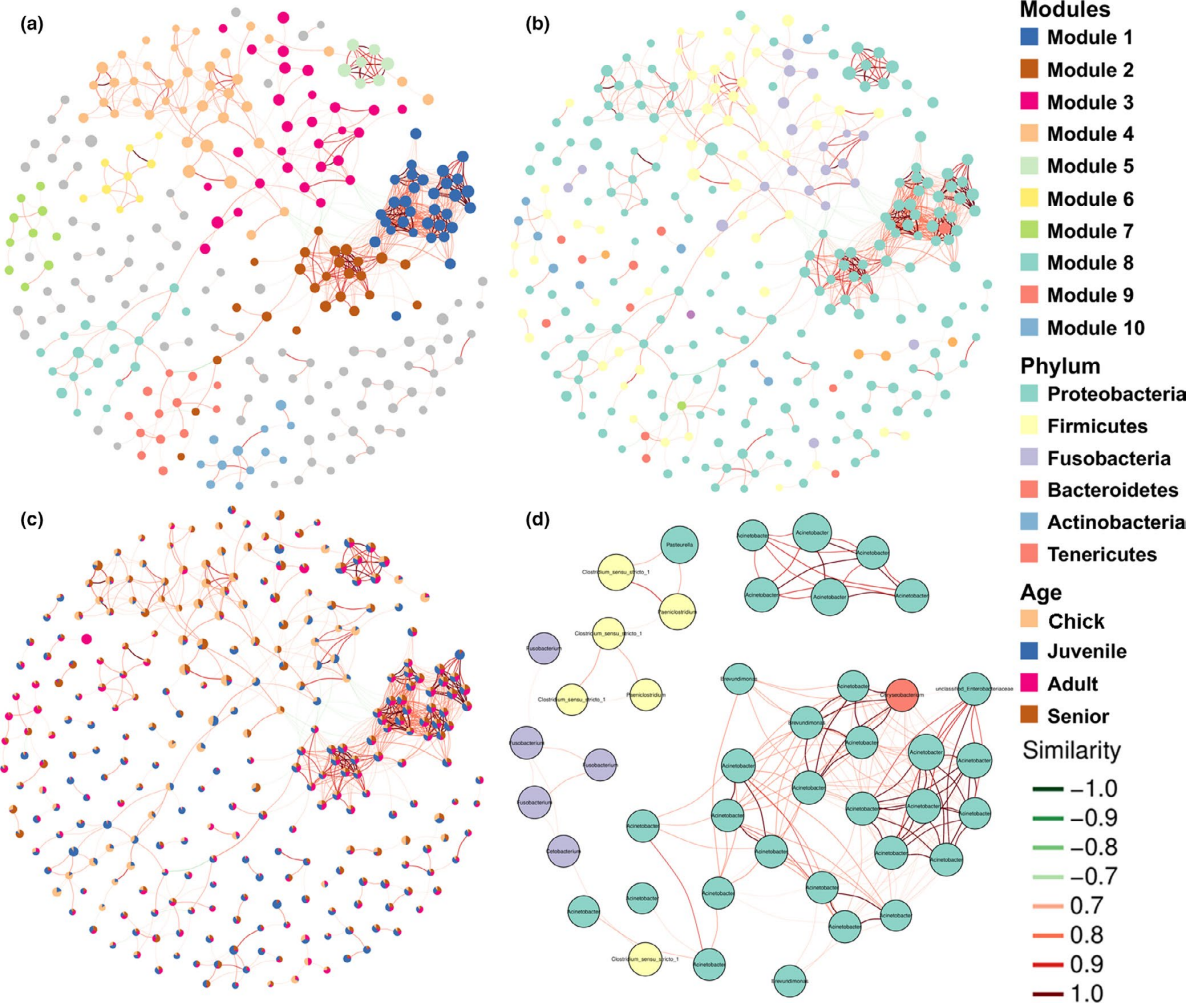
Co‐occurrence networks of total ASVs colored by modularity (a), taxonomy at the phylum level (b), and abundance ratio among different groups (c), and top 50 abundant ASVs (d)

We further investigated the age‐related changes in the abundance of various intestinal bacteria by constructing a co‐occurrence network using the ASV abundance table. The network had 281 nodes and 815 edges, with an average path length of 5.63, and clustered into 10 main modules (Figure 5a). Based on the taxonomy, most modules were occupied by *Proteobacteria*, ASVs in Module 3 were mainly *Firmicutes* and *Fusobacteria*, and part of Module 4 was dominated by *Firmicutes* (Figure 5b). Moreover, ASVs that had obvious more correlations with other ASVs (Module 1, 2, and 5) existed in all four age groups with a relatively similar abundance ratio, whereas ASVs that were only correlated with limited ASVs showed age‐related variations in abundance (Figure 5c). We also constructed a subnetwork using the top 50 abundant ASVs in all the studied samples. Multiple ASVs belonging to the phylum *Proteobacteria* and genus *Acinetobacter* showed complex correlations with each other, while several ASVs belonging to the phyla *Firmicutes* and *Fusobacteria* clustered alone and were separate from other ASVs (Figure 5d). These results indicated that the variations in gut microbiota in *P*. *antarctica* showed phylogenetic specificity, and more common bacteria might have closer interactions.

### 3.6 Variations in metabolic functions of gut microbiota

**FIGURE 6:**
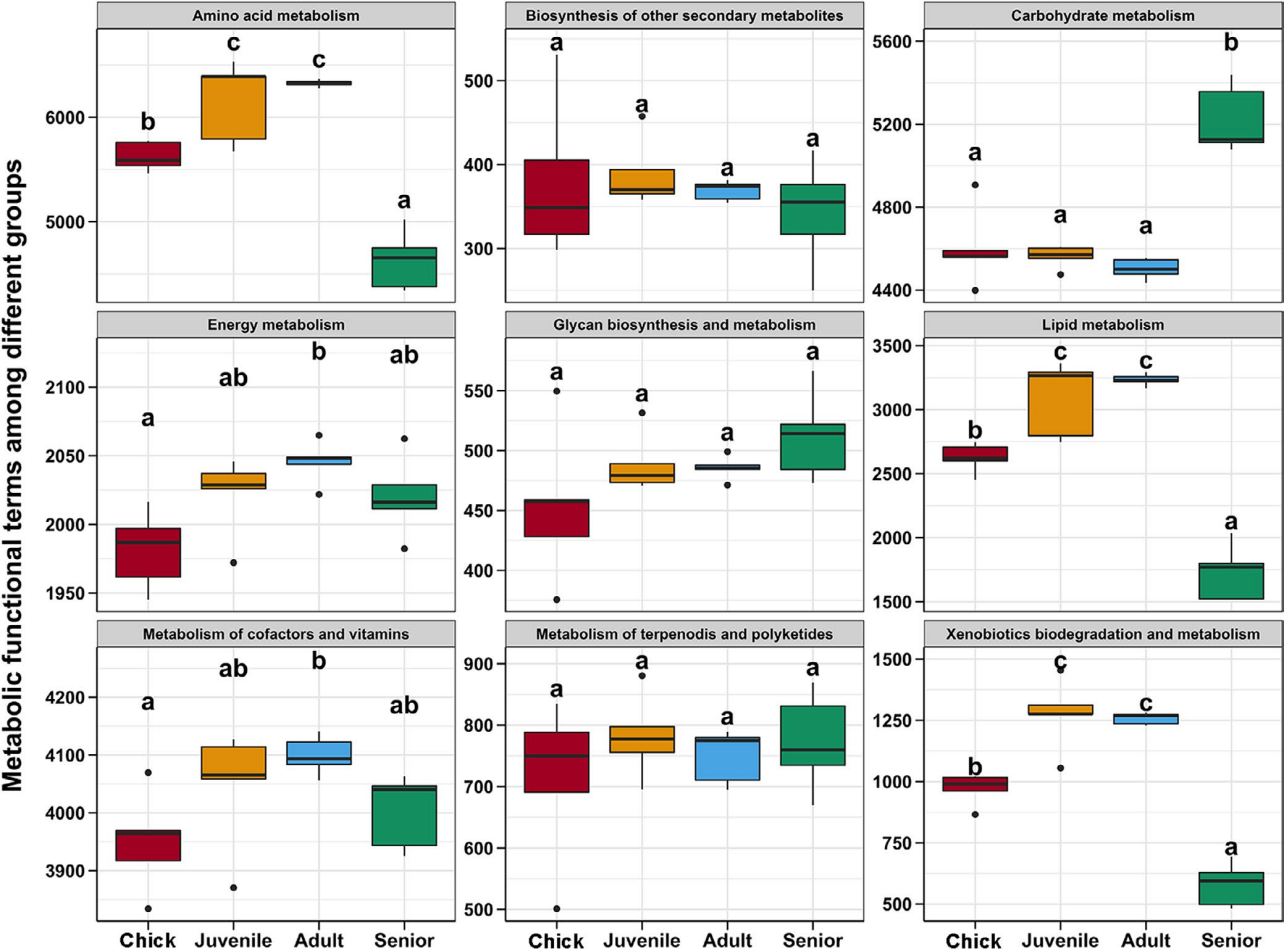
Age‐related differences in predicted metabolic functions of gut microbiota in *P*. *antarctica*. Different lowercase letters above each box in the same subfigure represent significant differences between groups (Tukey's HSD test, *p* < 0.05)

The predicted functions related to metabolism based on the KEGG database were used to clarify the age‐related differences in gut microbiota functions in *P*. *antarctica*. There were similar trends in the abundances of metabolic pathways related to the amino acid, energy, lipid, cofactors, vitamins, and xenobiotics, all of which increased from chicks to adults and then decreased in seniors (*p* < 0.05, Figure 6). There was no significant difference in the metabolism of secondary metabolites, glycans, terpenoids, and polyketides among the different age groups (*p* > 0.05, Figure 6). In contrast, carbohydrate metabolism pathways were significantly more abundant in senior birds compared with other ages (*p* < 0.05, Figure 6).

## 4 DISCUSSION

The composition structures of the gut microbiota have been reported for some species of penguins. Penguin gut microbiota was first reported by Banks et al. in 2009, who obtained 52 operational taxonomic units (OTUs) at 99% similarity using clone library technology and samples from several Adélie penguins. Dewar et al. (2013) compared the gut microbiota of four penguin species using pyrosequencing, and found clusters of 2,195, 1,362, 1,331, and 561 OTUs at 97% similarity in gentoo, macaroni, king, and little penguins, respectively. Two recent studies analyzed the composition of the gut microbiota in Chinstrap penguins using high‐throughput sequencing based on the bacterial 16S rRNA gene. Barbosa et al. (2016) retrieved 3,621 OTUs at 97% similarity in fecal samples from 53 wild Chinstrap penguins, and Lee et al. (2019) assigned the 16S rRNA gene sequences to 19,990 OTUs at 97% similarity level from 27 individuals. In this study, we investigated the composition of the gut microbiota in 20 Chinstrap penguins and obtained a total of 6,373 ASVs. This study uses the novel ASV clustering method to investigate the gut microbiota in Chinstrap penguins, which assigns sequences based on 100% similarity and can thus provide more accurate taxonomy and abundance results (Bokulich et al., 2018).

The absolute number of OTUs is not suitable for comparing the differences in the gut microbiota of penguins among studies due to the different numbers of sequences (Ley et al., 2008). We therefore compared the alpha diversity indices of gut microbiota between the present and previous studies. A previous study found average Chao1 and Shannon indices of 348 and 2.97, respectively, in the gut microbiota from wild Chinstrap adults and chicks (Barbosa et al., 2016), while another study found a lower Shannon index (1.5–3.0) in Chinstrap penguins during the feeding and molting stages (Lee et al., 2019). These values were much lower than those found in the present study, indicating that the gut microbiota in captive Chinstrap penguins showed greater richness, less dominance, and less variability compared with wild individuals.

Diet and the environment have been observed to be vigorous predictors of gut microbiota in diverse animals (Ley et al., 2008). For example, differences in the gut microbiota of Australian sea lions (*Neophoca cinerea*) could be contributing to the diverse environmental bacteria from seawater in different colonies (Delport et al., 2016). Moreover, the gut microbiota in whales and terrestrial carnivores exhibited similar amino acid synthesis and protein catabolism, while fermentative metabolism‐related microbes in the whale gut more closely resembled those of terrestrial herbivores (Sanders et al., 2015). However, such correlations are less clear in birds (Kohl, 2012). *Firmicutes* and *Fusobacteria* were found the dominant bacterial phyla in wild Chinstrap penguins (Barbosa et al., 2016; Lee et al., 2019), while the present study has identified *Proteobacteria* as the most abundant bacterial phylum in captive Chinstrap penguins. This difference may relate to the different diets of wild and captive Chinstrap penguins: In their natural environment, Chinstrap penguins tend to specialize in Antarctic krill (Herman et al., 2017), while captive individuals were fed on fish. Although the protein and fat contents of the two food items are similar, the bacteria that degrade these foods might be significantly different. Moreover, captive Chinstrap penguins live in an artificial environment with limited environmental microbes, which might also restrict the acquisition of new additions to the gut microbiota.

The current study demonstrated age‐related shifts in the diversity and composition of the gut microbiota in Chinstrap penguins. In particular, the gut microbiota of *P*. *antarctica* might decline after maturity, being most stable during adulthood. Given that chicks are fed exclusively on food redigested by the parents, their immature gut microbiota might be related to the reduced requirements of digestion capabilities. In human beings, the gut microbiota changes after the introduction of solid food and becomes adult‐like after 1‐year old (Vallès et al., 2012). In little penguins, the abundance of *Firmicutes* and *Bacteroidetes* was observed with an upward trend throughout their development from chicks to adults (Dewar et al., 2017). In mammals, the microbial communities are inherited from the mother through contact with their fecal and vaginal microbes and from breast milk (Palmer et al., 2007). In birds, however, newly hatched chicks acquire their microbiota from multiple sources, including the surface of the egg, the surrounding environment (i.e., nest), and their first meal (Lucas & Heeb, 2005; Rehman et al., 2007). A study of little penguins and short‐tailed shearwaters indicated that the adult's microbiota may have a negligible influence over the chick's microbiota, while differences in diet composition and digestive physiology could be more important (Dewar et al., 2017). Similarly, Chinstrap penguins might be expected to mature their gut microbiota after their diet becomes self‐supplied.

The present study also revealed that variations in the relative abundances of few dominant bacteria might be the main contributor to the differences in the gut microbiota of *P*. *antarctica* with different ages. Analyses of the core microbiota and co‐occurrence network demonstrated that the key bacteria included *Acinetobacter*, *Clostridium* sensu stricto, and *Fusobacterium*. The genus of *Acinetobacter* is a Gram‐negative bacterium belonging to the class of *Gammaproteobacteria*, some species of which are responsible for infections in debilitated patients in hospitals (Dent Lemuel et al., 2010). However, the genus *Acinetobacter* was dominant in the gut microbiota in Chinstrap penguins, especially in adults, suggesting that some species of *Acinetobacter* are not pathogenic, and play an important role in food metabolism, at least in Chinstrap penguins. In contrast, *Fusobacterium* was more abundant in some individuals from chicks to seniors. *Fusobacterium* is a Gram‐negative, non‐spore‐forming bacterium, widely accepted as an animal and human pathogen (Castellarin et al., 2012). More abundant *Fusobacterium* was found in oral tissues from diseased striped dolphins compared to the healthy individuals (Godoy‐Vitorino et al., 2017). Moreover, *Fusobacterium* has been found as the potential pathogen responsible for diarrhea in spotted seals (Tian, Du, Han, Wang, et al., 2020). This suggests that Chinstrap penguins may be more susceptible to infections during the chick and senior stages.

Finally, the PICRUSt2 results showed the changes in functional pathways of the gut microbiota during aging in Chinstrap penguins. Most metabolic pathways showed trends in line with the gut microbiota compositions, being more abundant in adults and less abundant in chicks and seniors. However, carbohydrate metabolism pathways were significantly more abundant in senior birds compared with the other ages. This result suggests that more carbohydrates should be added to the diet for older Chinstrap penguins, to maintain their energy supply. Moreover, all studies of the gut microbiota in penguins have utilized the 16S rRNA gene sequence and prediction of functions. Therefore, metagenomic analysis is required to illustrate the function of the gut microbiota in penguins during different physiological processes.

## 5 CONCLUSIONS

In summary, we used fecal sampling and high‐throughput sequencing to determine the age‐related differences in the gut microbiota of Chinstrap penguins. The diversity and composition of the gut microbiota in captive Chinstrap penguins appears to change with age, largely due to changes in key bacteria such as *Acinetobacter* and *Fusobacterium*. The gut microbiota of Chinstrap penguins was most stable during adulthood, followed by a sharp decline. The predicted functions of the gut microbiota provide a valuable perspective on age‐related changes in food metabolism in Chinstrap penguins. However, further metagenomics studies are required to verify these conclusions in captive Chinstrap penguins.

## CONFLICT OF INTEREST

None declared.

## AUTHOR CONTRIBUTIONS

**Jiashen Tian:** Conceptualization (equal); Formal analysis (equal); Methodology (equal); Resources (equal); Writing‐original draft (equal). **Jing Du:** Conceptualization (equal); Supervision (equal); Writing‐review & editing (equal). **Shengjiu Zhang:** Resources (equal). **Yanqiu Li:** Resources (equal). **Gao Xianggang:** Formal analysis (equal); Investigation (equal). **Jiabo Han:** Conceptualization (equal); Supervision (equal); Writing‐review & editing (equal). **Zhichuang Lu:** Writing‐review & editing (equal).

## ETHICS STATEMENT

This study has been authorized by the Dalian Sun Asia Aquarium. No animals were directly involved.

## Data Availability

Raw sequence reads are available at the National Center for Biotechnology Information's Sequence Read Archive under the accession number PRJNA704214: https://www.ncbi.nlm.nih.gov/bioproject/PRJNA704214.
